# Hyponatremia Improvement Is Associated with a Reduced Risk of Mortality: Evidence from a Meta-Analysis

**DOI:** 10.1371/journal.pone.0124105

**Published:** 2015-04-23

**Authors:** Giovanni Corona, Corinna Giuliani, Joseph G. Verbalis, Gianni Forti, Mario Maggi, Alessandro Peri

**Affiliations:** 1 Endocrinology Unit, Maggiore-Bellaria Hospital, Bologna, Italy; 2 Endocrine Unit, “Center for Research, Transfer and High Education on Chronic, Inflammatory, Degenerative and Neoplastic Disorders for the Development of Novel Therapies” (DENOThe), Department of Experimental and Clinical Biomedical Sciences “Mario Serio”, University of Florence, Careggi Hospital, 50139, Florence, Italy; 3 Division of Endocrinology and Metabolism, Georgetown University, Washington, DC, 20007, United States of America; 4 Andrology Unit, “Center for Research, Transfer and High Education on Chronic, Inflammatory, Degenerative and Neoplastic Disorders for the Development of Novel Therapies” (DENOThe), Department of Experimental and Clinical Biomedical Sciences “Mario Serio”, University of Florence, Careggi Hospital, 50139, Florence, Italy; University of Florida, UNITED STATES

## Abstract

**Background:**

Hyponatremia is the most common electrolyte disorder and it is associated with increased morbidity and mortality. However, there is no clear demonstration that the improvement of serum sodium concentration ([Na^+^]) counteracts the increased risk of mortality associated with hyponatremia. Thus, we performed a meta-analysis that included the published studies that addressed the effect of hyponatremia improvement on mortality.

**Methods and Findings:**

A Medline, Embase and Cochrane search was performed to retrieve all English-language studies of human subjects published up to June 30^th^ 2014, using the following words: “hyponatremia”, “hyponatraemia”, “mortality”, “morbidity” and “sodium”. Fifteen studies satisfied inclusion criteria encompassing a total of 13,816 patients. The identification of relevant abstracts, the selection of studies and the subsequent data extraction were performed independently by two of the authors, and conflicts resolved by a third investigator. Across all fifteen studies, any improvement of hyponatremia was associated with a reduced risk of overall mortality (OR=0.57[0.40-0.81]). The association was even stronger when only those studies (n=8) reporting a threshold for serum [Na^+^] improvement to >130 mmol/L were considered (OR=0.51[0.31-0.86]). The reduced mortality rate persisted at follow-up (OR=0.55[0.36-0.84] at 12 months). Meta-regression analyses showed that the reduced mortality associated with hyponatremia improvement was more evident in older subjects and in those with lower serum [Na^+^] at enrollment.

**Conclusions:**

This meta-analysis documents for the first time that improvement in serum [Na^+^] in hyponatremic patients is associated with a reduction of overall mortality.

## Introduction

Hyponatremia is the most common electrolyte disorder encountered in clinical practice, and in its mild presentation (i.e. serum [Na^+^] 130–134 mmol/L) occurs in up to 30% of hospitalized patients [1, 2]. It is well known that acute severe hyponatremia may have dramatic consequences caused by cerebral edema and may lead to death [3]. There is now convincing evidence that also mild chronic hyponatremia, traditionally not considered as a potentially harmful condition, may actually be associated with adverse effects, such as gait alterations and falls, attention deficits [4], bone loss and fractures [5–8]. Interestingly, it has been demonstrated that chronic hyponatremia exacerbates multiple signs of senescence in aged rats, such as sarcopenia, osteoporosis, cardiac fibrosis, and hypogonadism [9]. These manifestations are somewhat unexpected, considering that in chronic hyponatremia adaptation mechanisms should establish a new osmotic equilibrium across the plasma membrane; consequently, water moves out of the cells, thus reducing brain edema. However, experimental data showed that low extracellular [Na^+^] directly affects cell homeostasis in neuronal cells as well as in osteoclast precursors, independently of reduced osmolality [8, 10]. Therefore, these data demonstrated that the detrimental effects of hyponatremia extend beyond the “osmotic theory”, in agreement with clinical observations.

A number of studies have reported an association between hyponatremia, even when mild to moderate, and mortality across many diverse conditions (e.g., pneumonia, heart failure, acute myocardial infarction, cirrhosis and cancer [11–16]). We have recently reported an extensive meta-analysis to assess the relationship between hyponatremia and mortality. Data from 81 studies for a total of 147,948 hyponatremic subjects indicated that hyponatremia is associated with an increased risk of overall mortality (RR = 2.60[2.31–2.93]) [17]. Furthermore, hyponatremia appeared associated with an increased risk of death when patients were analyzed separately based on specific diseases, such as myocardial infarction, heart failure, cirrhosis and pulmonary infections. Interestingly, a quite small difference in serum [Na^+^] (mean 4.8 mmol/L) was detected in patients who died compared to survivors.

Whether the improvement of hyponatremia counteracts the increased risk of mortality associated with hyponatremia has not been clearly ascertained, so far. In the Efficacy of Vasopressin Antagonism in Heart Failure Outcome Study with Tolvaptan (EVEREST) study, neither all-cause nor cardiovascular mortality significantly differed between patients with heart failure treated with the vasopressin type 2 receptor antagonist, tolvaptan, and those receiving placebo [18]. However, a subset analysis of those patients in EVEREST who were markedly hyponatremic (defined as a serum [Na^+^] <130 mEq/L) did show a significant reduction of cardiovascular morbidity and mortality after discharge in patients treated with tolvaptan [19]. Nonetheless, EVEREST was not powered to examine outcomes in the smaller subgroup of patients enrolled who had both heart failure and hyponatremia. A significant positive relationship between an increase in serum [Na^+^] and decreased mortality was found in 322 patients hospitalized for acute heart failure and followed for up to 3 years [20]. Conversely, a multicenter analysis of almost 3000 patients hospitalized for acute heart failure in Korea showed that patients admitted with hyponatremia had a worse prognosis compared to those with normonatremia, but this relation persisted regardless of whether the hyponatremia improved during the hospitalization [21]. However, this study presented some limitations, because it was a retrospective analysis from a registry and not a prospective randomized trial. In addition, the change in serum [Na^+^] was assessed only once, prior to or at discharge from the hospital. The aim of this study was to perform a meta-analysis based on published studies of hyponatremic patients that included data on the effect of correction of serum [Na^+^] on mortality.

## Methods

This meta-analysis was performed according to the Preferred Reporting Items for Systematic Reviews and Meta-Analyses (PRISMA) checklist (S1 File) (http://www.prisma-statement.org/).

### Eligibility criteria

Studies specifically addressing the association between mortality rate and improved or normalized serum [Na^+^] were included in the analysis

### Information source and Search strategy

An extensive Medline, Embase, and Cochrane search was performed including the following words: ("hyponatraemia"[All Fields] OR "hyponatremia"[MeSH Terms] OR "hyponatremia"[All Fields]) AND ("mortality"[Subheading] OR "mortality"[All Fields] OR "mortality"[MeSH Terms]) AND ("epidemiology"[Subheading] OR "epidemiology"[All Fields] OR "morbidity"[All Fields] OR "morbidity"[MeSH Terms]) AND ("sodium, dietary"[MeSH Terms] OR ("sodium"[All Fields] AND "dietary"[All Fields]) OR "dietary sodium"[All Fields] OR "sodium"[All Fields] OR "sodium"[MeSH Terms]). The search up to June 30^th^ 2014 was restricted to English-language articles and studies of human participants. A hand-searched bibliography of retrieved papers for additional references was performed. Details of the literature search process are outlined in the flow chart. The identification of relevant abstracts, the selection of studies based on the criteria described above, and the subsequent data extraction were performed independently by two of the authors (G.C., C.G.), and conflicts resolved by a third investigator (A.P.).

### Study selection

**Fig 1:**
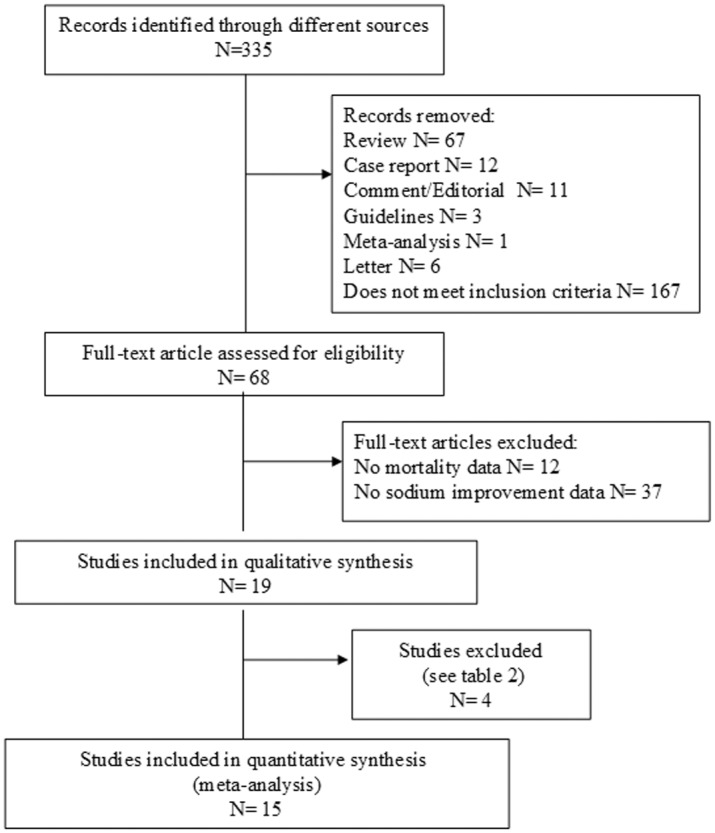
Trial flow diagram.

The meta-analysis was performed including all studies comparing mortality rate in subjects with or without improvement of hyponatremia (see Fig 1 and Table 1). Studies not specifically addressing the association between mortality rate and improved or normalized serum [Na^+^] were excluded from the analysis (see Table 2).

**Table 1:** Observational studies included in the meta-analysis.

Source	Type of disease	Age (years)	Male %	Na^+^ cut-off (mEq/L)	Patients (n)	Persistent HN (n)	Improved HN (n)	Deaths Persistent HN (n)	Deaths Improved HN (n)
Licata et al., 2003 [25]	HF	76.4	63.5	135	107	54	53	43	20
Klein et al, 2005 [13]	HF	68	68	135	244	151	93	11	12
Hoorn et al, 2006 [2]	Hospitalized	60.9+16.9	48.7	125	74	19	55	7	7
Gheorghiade et al, 2007 [26]	HF	53,6	NA	134	103	71	32	22	9
Rossi et al, 2007 [27]	HF	65	67	135	68	23	45	5	5
Hackworth et al, 2009 [28]	Liver transplantation	51.7+7.8	80.7	130	90	34	56*	2	8
Rusinaru et al, 2009 [29]	HF	77.3+9.2	46	136	91	46	45*	42	33
Waikar et al, 2009 [14]	Hospitalized	67	NA	NA	8318	4524	3794	1846	1461
Hansen et al, 2010 [30]	SCLC	NA	NA	NA	61	46	15*	NA	NA
Madan et al, 2011 [20]	HF	65.9+15.8	55.2	135	279	57	222	53	208
Lee et al, 2012 [21]	HF	70.5	NA	135	464	190	274*	185	263
Vaishya et al, 2012 [31]	Emergency in patients	NA	58.2	120	175	106	69*	64	27
Ng et al, 2013 [32]	APE	73.5+12.7	48	135	114	56	58*	55	56
Qureshi et al, 2013 [33]	MI	67.5+16.4	55.7	134	1798	280	1518*	155	425
Darmon et al, 2014 [34]	Intensive care unit patients	63.9	60.6	135	1830	811	1019	163	179

* = threshold for serum [Na^+^] improvement >130 mmol/L.

HN: hyponatremia; HF: heart failure; SCLC: small cell lung cancer; APE: acute pulmonary embolism; MI; myocardial infarction.

**Table 2:** Studies that met inclusion criteria but did not provide data for meta-analysis.

First author, year	Brief description of the study and main conclusions
Nzerue et al, 2003 [35]	Retrospective study of 168 hospitalized patients treated for severe hyponatremia. Mortality was higher in patients with slow correction rate but there were no data about mortality rate in patients with corrected or improved hyponatremia *vs* patients with persistent hyponatremia.
Doshi et al, 2012 [36]	Retrospective analysis of 4702 hospitalized patients with cancer, of which 47% were hyponatremic. Increase in serum sodium was associated with lower 90-day mortality, but there were no data about mortality rate in patients with corrected or improved hyponatremia *vs* patients with persistent hyponatremia.
Hauptman et al, 2013 [19]	Analysis of data from the EVEREST trial [18] to assess the clinical course and the outcomes of hospitalized patients with heart failure and hyponatremia and treated with tolvaptan. In patients with severe hyponatremia, tolvaptan therapy was associated with reduced cardiovascular mortality but it was not specified whether serum [Na^+^] was normalized or improved.
Lee et al, 2013 [37]	Retrospective review of the electronic medical records of 512 patients who received a liver transplant, of which 48% were hyponatremic. Delta sodium concentrations were associated with a higher in-hospital mortality, but were no data about mortality rate in patients with corrected, improved or overcorrected hyponatremia *vs* patients with persistent hyponatremia

### Outcome and quality assessment

The principal outcome of this analysis was the effect of serum [Na^+^] improvement (whatever obtained), on mortality rate. A secondary outcome included the effect of serum [Na^+^] >130 mmol/L at follow-up on mortality rate. The quality of the studies was assessed using the Cochrane criteria [22].

### Statistical analysis

Heterogeneity on mortality rate was assessed by using I^2^ statistics. Even when a low heterogeneity was detected, a random-effects model was applied, because the validity of tests of heterogeneity can be limited with a small number of component studies.

**Fig 2:**
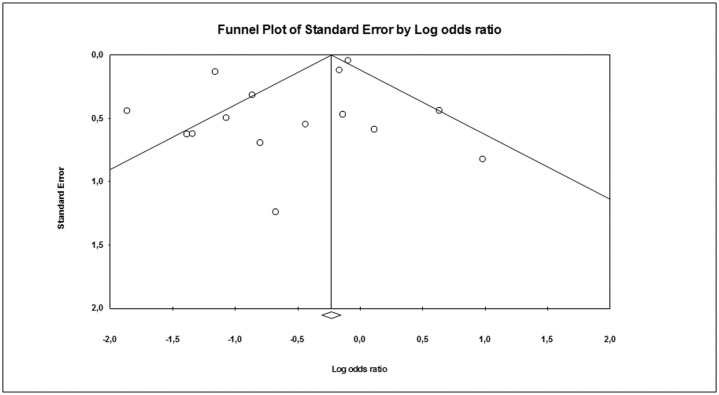
Funnel plot of the observational studies included in the meta-analysis.

In order to estimate possible publication or disclosure bias, we used funnel plots, the Begg adjusted rank correlation test and Egger's [23, 24] (Fig 2). However, because these tests have low statistical power when the number of trials is small, undetected bias may still be present. Odds ratio with 95% Confidence Interval (OR) was calculated for mortality rate after sodium improvement. A sensitivity analysis was performed considering those studies reporting data on mortality rate after serum [Na^+^] improvement at 12 and 36 months of follow-up or after serum [Na^+^] ≥130 mmol/L at follow-up. A meta-regression analysis was performed to test the effect of age and serum [Na^+^] cut-off enrolment on overall mortality rate. In addition, a linear regression analysis model, weighting each study for the number of subjects enrolled, was performed to verify the independent effect of hyponatremia improvement on mortality after the adjustment for age and sex and serum [Na^+^] cut-off.

All data were calculated using Comprehensive Meta-analysis Version 2, Biostat, and (Englewood, NJ, USA). Logistic multivariate analysis was performed on SPSS (Statistical Package for the Social Sciences; Chicago, USA) for Windows 20.1.

## Results

Out of 335 retrieved articles, 320 articles were excluded for different reasons. The flow of the meta-analysis is summarized in Fig 1, and the characteristics of the trials included in the meta-analysis are summarized in Table 1 [2, 13, 14, 20, 21, 25–34]. Sixty-eight full-text articles were considered potentially eligible for the meta-analysis. However, 49 of them were excluded because they did not included information about serum [Na^+^] improvement (n = 37) or about mortality (n = 12) (see S2 file). Four additional studies were excluded because they did not specifically address the association between mortality rate and serum [Na^+^] improvement or normalization (Table 2) [19, 35–37]. Among the 15 selected studies, 7 studies evaluated the effect of hyponatremia improvement on overall mortality rate in subjects with heart failure, 2 in hospitalized series of subjects, one each including patients subjected to orthopic liver transplantation, admitted to an emergency room or to an intensive care unit, affected by lung cancer, myocardial infarction or pulmonary acute embolism (Table 1). All the studies included in the analysis were observational ones.

Overall, 13,816 hyponatremic subjects with a mean age of 66.1 years and a mean follow-up of 33.6 months were included in the meta-analysis. Hyponatremia was defined according to varying cut-off definitions in the included studies (Table 1). In particular, 14 studies enrolled only hyponatremic subjects, whereas in one case [25] a mixed cohort of hyponatremic/eunatremic patients was considered. Among the studies that were evaluated, an improvement of serum [Na^+^] of any degree was obtained in 53.2% of patients through different approaches. However, about 1 out of 4 patients (27%) did not reach a threshold of 130 mmol/L, after improvement (Table 1). I^2^ was 85.5 (p<0.0001) and the Begg-adjusted rank correlation test, calculated on the basis of overall mortality rate for hyponatremia improvement, suggested no major publication bias (Kendall tau 0.00; p = 1.0). Similar results were obtained by applying Egger’s regression (Intercept -1.24±0.79; p = 0.14).

**Fig 3:**
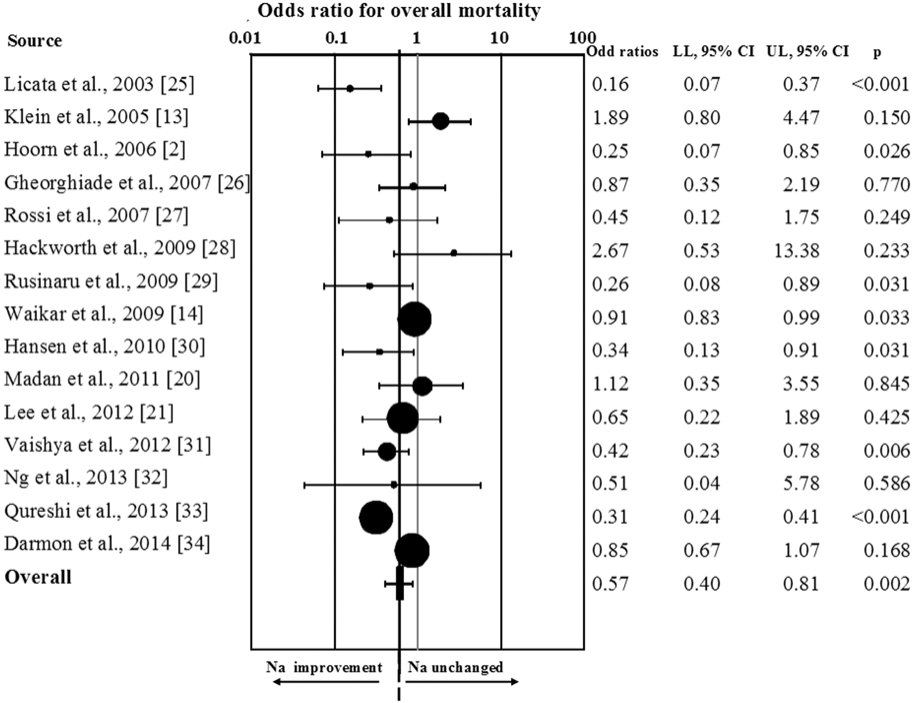
Odds ratio for overall mortality rate in patients with any increase of serum [Na^+^].

**Fig 4:**
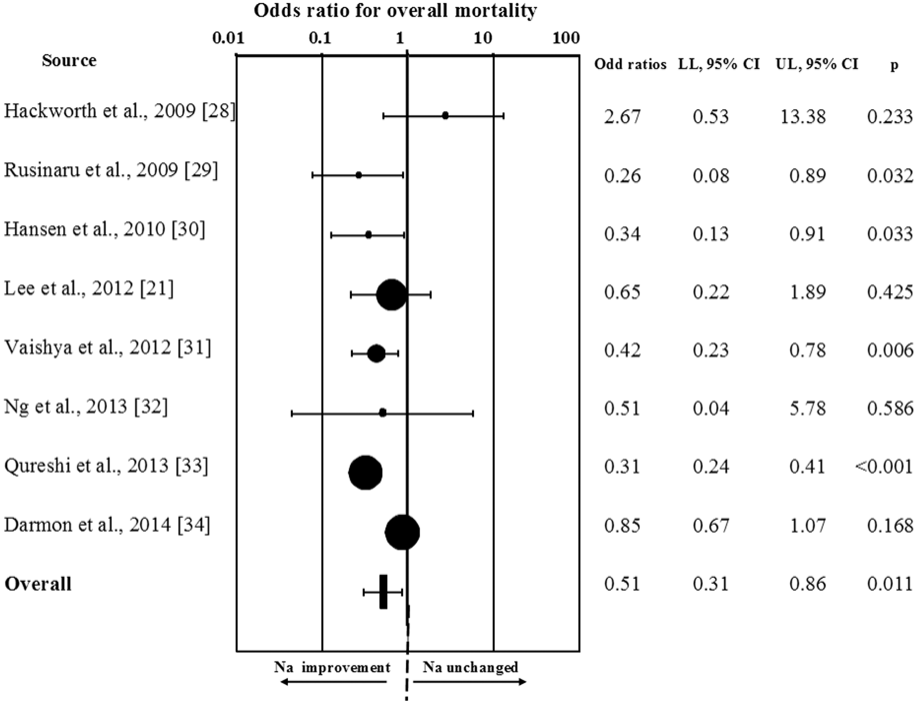
Odds ratio for overall mortality rate in patients from studies in which a threshold for serum [Na^+^] improvement >130 mmol/L was reported.

When all studies were considered, hyponatremia improvement was significantly associated with a reduction of overall mortality (OR = 0.57[0.40;0.81]; p = 0.002; Fig 3). Similar results were observed when the study enrolling a mixed population of hyponatremic/eunatremic subjects was excluded from the analysis (OR = 0.63[0.44;0.89]; p = 0.001). Interestingly, the results were even more impressive by performing a sensitivity analysis, which considered only those studies (n = 8) reporting a threshold for serum [Na^+^] improvement >130 mmol/L (RR = 0.51[0.31;0.86]; p<0.001; (Fig 4). The favorable effect of hyponatremia improvement on mortality rate was confirmed in those studies (n = 4) reporting data at 12 months of follow-up (OR = 0.55[0.36;0.84];p = 0.006), and a trend toward a reduction of mortality rate was observed at 36 months (n = 3, OR = 0.67[0.45;1.02];p = 0.06).

Interestingly, a meta-regression analysis showed that the reduced mortality rate in patients in whom hyponatremia improved was more evident in older subjects (S = −0.06[−0.010;−0.02];p = 0.004;I = 3.62[1.01;6.24];p = 0.01) and in those with lower serum [Na^+^] at enrollment (S = 0.07[0.03;0.001];p = 0.001; I = −9.10[−14.37;−3.84];p = 0.001). The association between reduced mortality rate after serum [Na^+^] improvement and [Na^+^] cut-off at enrollment was confirmed in a multiple regression model even after adjustment for age, gender and follow-up (adj.r = 0.39; p<0.0001).

## Discussion

There is evidence from the literature that hyponatremia is associated with an increased risk of mortality in patients with diverse clinical conditions, and in a recent meta-analysis we definitively confirmed this finding [17]. On the other hand, it has not been clearly established, so far, whether an improvement of hyponatremia is able to revert or decrease the increased risk of death associated with hyponatremia.

Therefore, we performed a meta-analysis, in which we included all of the English-language published studies until June 30^th^ 2014 that compared the mortality rate in human subjects with or without interval improvement of hyponatremia. Fifteen published studies were selected according to the specified inclusion criteria yielding a total of 13,816 hyponatremic patients with a mean follow-up of 33.6 months. We found that an increase of serum [Na^+^] was obtained in about half of the patients. This finding in principle suggests that the management of patients with hyponatremia is far from being optimal, in agreement with data from the literature [38, 39]. In those patients in whom serum [Na^+^] was improved, the overall mortality rate was reduced up to 60% compared to patients with no improvement of their hyponatremia. These data were obtained by considering any increase of serum [Na^+^]. However, the association between improvement of hyponatremia and reduced mortality was even stronger when we performed a sensitivity analysis based on those studies in which a threshold for improvement of at least 130 mmol/L was reported (up to 70% of mortality rate reduction); this result increases the strength of the relationship between significant increases in serum [Na^+^] and reduction of mortality rate.

We also observed that the reduced risk of mortality associated with hyponatremia improvement appears to last during prolonged follow-up. Specifically, a significantly reduced mortality persisted at 12 months of follow-up, and a similar trend was observed al 36 months. In the latter case, the absence of a statistically significant difference may very likely be due to the smaller number of studies in which a more prolonged follow-up was performed [14, 29, 32]. It might be also hypothesized that other factors might mitigate the effect of serum [Na^+^] improvement on mortality rate in the long term.

Another interesting finding from a meta-regression analysis was that the effect of the improvement of hyponatremia on the reduced risk of mortality increases as a function of the prevalence of patients with a more advanced age. This observation is of particular importance because hyponatremia occurs more commonly in elderly subjects [1]. Another meta-regression analysis also indicated that the beneficial effect of the improvement of hyponatremia is more evident in patients with lower serum [Na^+^] at enrollment. This finding implies that these patients deserve an even more thoughtful workup for the correction of hyponatremia, because the outcome may be dramatically more favorable compared to patients not properly treated.

Overall, this meta-analysis demonstrates that an improvement in serum [Na^+^] is associated with a decreased mortality rate observed in hyponatremic patients, although a cause-effect relationship cannot be extrapolated from the studies that have been analyzed. In addition, some limitations of our meta-analysis must be recognized. First of all, the present data cannot clarify whether hyponatremia and/or its lack of correction contribute directly to poor outcomes or are simply markers for severity of underlying co-morbidities, and whether improvements of the underlying co-morbidities influenced both the serum [Na^+^] and the mortality of the hyponatremic patients [40]. Accordingly, it should recognized that the data were adjusted only for age and gender, whereas the prevalence of associate morbidities was not considered as possible confounders, because they were not reported adequately in a sufficient number of studies. Hence, potential unmeasured confounders may have caused residual confounding effects, but the measured factors that are correlated with such confounders should have mitigated this bias. However, meta-analysis is particularly useful when there is a variety of reports with low statistical power; in this situation, pooling of data can improve power and provide a more convincing result. It should be also recognized that the duration of the available trials is relatively short. A further limitation is represented by incomplete reporting of the data on mortality rate in trials only marginally designed for the assessment of mortality endpoints after [Na^+^] improvement. In particular, all the data that were analyzed were from observational studies and none of them was originally designed to address clinical outcomes after serum [Na^+^] improvement. Accordingly, no placebo-controlled studies were available for inclusion in the present meta-analysis. Finally, the statistical analysis showed the presence of heterogeneity that was particularly evident in two studies [13, 28]. Finally, statistical analyses did not suggest any relevant publication bias, the possibility of selective reporting cannot be excluded. These considerations do not negate the value of the novel findings reported in our study, but rather highlight the need for additional, well-designed studies of clinical outcomes with effective therapies in patients with hyponatremia [41–43].

## Supporting Information

S1 File Prisma Checklist. (DOC)

S2 File Full-text articles that were excluded from the analysis. (DOC)
