# Correction: Hyponatremia Improvement Is Associated with a Reduced Risk of Mortality: Evidence from a Meta-Analysis

**DOI:** 10.1371/journal.pone.0152846

**Published:** 2016-03-28

**Authors:** Giovanni Corona, Corinna Giuliani, Joseph G. Verbalis, Gianni Forti, Mario Maggi, Alessandro Peri

There are errors in Fig 3, “Odds ratio for overall mortality rate in patients with any increase of serum [Na^+^].” Please see the corrected Fig 3 here.

**Fig 3:**
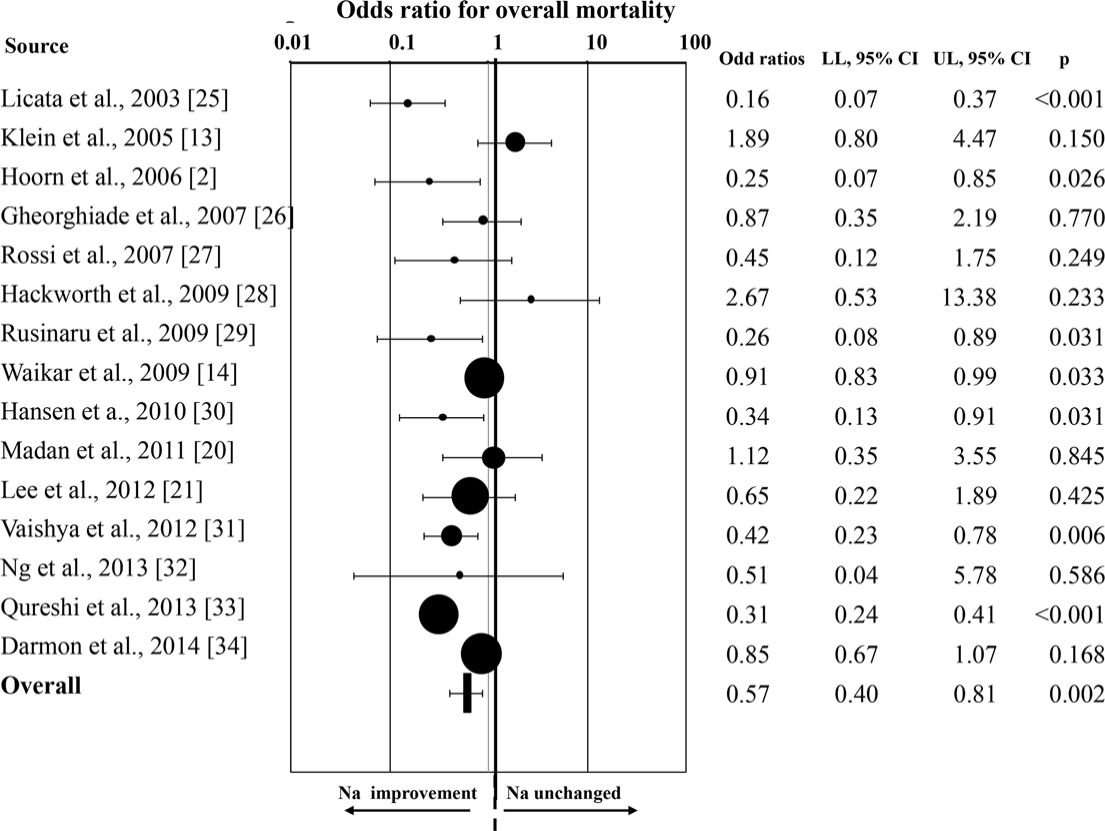
Odds ratio for overall mortality rate in patients with any increase of serum [Na^+^].
