# Supplementary material for: Hyponatremia Improvement Is Associated with a Reduced Risk of Mortality: Evidence from a Meta-Analysis
Source: PLoS One. 2015 Apr 23;10(4):e0124105. doi: 10.1371/journal.pone.0124105 (PMC4408113; doi:10.1371/journal.pone.0124105)
Supplement: S1 File — (DOC) [file pone.0124105.s001.doc]

| **Section/topic** | **#** | **Checklist item** | **Reported on page #** |
| --- | --- | --- | --- |
| **TITLE** | | |  |
| Title | 1 | **HYPONATREMIA IMPROVEMENT IS ASSOCIATED WITH A REDUCED RISK OF MORTALITY: EVIDENCE FROM A META-ANALYSIS** | 1 |
| **ABSTRACT** | | |  |
| Structured summary | 2 | Background: Hyponatremia is the most common electrolyte disorder and it is associated with increased morbidity and mortality. However, to date there is no clear demonstration that the improvement of serum sodium concentration ([Na+]) counteracts the increased risk of mortality associated with hyponatremia. The aim of our study was to perform a meta-analysis that included the published studies that addressed the effect of hyponatremia improvement on mortality.  Methods: A Medline, Embase and Cochrane search was performed to retrieve all English-language studies of human subjects published up to June 30th 2014, using the following words: “hyponatremia”, “mortality”, “morbidity” and “sodium”. Fiftteen studies satisfied inclusion criteria encompassing a total of 12,797 patients. The identification of relevant abstracts, the selection of studies and the subsequent data extraction were performed independently by two of the authors, and conflicts resolved by a third investigator.  Results: Across all fifteen studies, any improvement of hyponatremia improvement was significantly associated with a reduced risk of overall mortality (OR=0.59[0.41-0.85]). The association was even stronger when only those studies (n=8) reporting a threshold for serum [Na+] improvement to >130 mmol/L were considered (OR=0.40[0.28-0.59]). The reduced mortality rate persisted at follow-up (OR=0.51[0.36-0.84] at 12 months). Meta-regression analyses showed that the reduced mortality associated with hyponatremia improvement was more evident in older subjects and in those with lower serum [Na+] at enrollment.  Conclusions: This meta-analysis documents for the first time that improvement in serum [Na+] in hyponatremic patients is associated with a reduction in the increased mortality associated with this common electrolyte disorder. This finding strengthens recommendations to properly assess and more effectively treat hyponatremic patients. | 2 |
| **INTRODUCTION** | | |  |
| Rationale | 3 | Hyponatremia is associated with increased morbidity and mortality. However, to date there is no clear demonstration that the improvement of serum sodium concentration ([Na+]) counteracts the associated-increased risk of mortality | 4 |
| Objectives | 4 | We performed an updated systematic review and meta-analysis including the published studies that addressed the effect of hyponatremia improvement on mortality. | 4 |
| **METHODS** | | |  |
| Protocol and registration | 5 | The meta-analysis was performed including all studies comparing mortality rate in subjects with or without improvement of hyponatremia | 5 |
| Eligibility criteria | 6 | Studies not specifically addressing the association between mortality rate and improved or normalized serum [Na+] were excluded from the analysis (see Table 2). | 5 |
| Information sources | 7 | PubMed from 1965 – June 30, 2014  EMBASE from 1974 – June 30, 2014  Cochrane from 1967 – June 30, 2014.  The principal source of information was derived from published articles. | 5 |
| Search | 8 | An extensive Medline, Embase, and Cochrane search was performed including the following words: ("hyponatraemia"[All Fields] OR "hyponatremia"[MeSH Terms] OR "hyponatremia"[All Fields]) AND ("mortality"[Subheading] OR "mortality"[All Fields] OR "mortality"[MeSH Terms]) AND ("epidemiology"[Subheading] OR "epidemiology"[All Fields] OR "morbidity"[All Fields] OR "morbidity"[MeSH Terms]) AND ("sodium, dietary"[MeSH Terms] OR ("sodium"[All Fields] AND "dietary"[All Fields]) OR "dietary sodium"[All Fields] OR "sodium"[All Fields] OR "sodium"[MeSH Terms]) | 5 |
| Study selection | 9 | We did not employ a search software. We hand-searched bibliographies of retrieved papers for additional references  Details of the literature search process are outlined in the flow chart (Figure 1). | 5 |
| Data collection process | 10 | Data extraction were performed independently by independently by two of the authors (G.C., C.G.), and conflicts resolved by a third investigator (A.P.). The credentials of all investigators are indicated in the author list. | 5-6 |
| Data items | 11 | The principal outcome of this analysis was the effect of serum [Na+] improvement (whatever obtained), on mortality rate. A secondary outcome included the effect of serum [Na+] >130 mmol/L at follow-up on mortality rate. | 6 |
| Risk of bias in individual studies | 12 | Quality of the studies was assessed using the Cochrane criteria. | 5 |
| Summary measures | 13 | The identification of relevant studies was performed independently by two of the authors (G.C., C.G.), and conflicts resolved by a third investigator (A.P.). Odds ratio with 95% Confidence Interval (OR) was calculated for mortality rate after sodium improvement. | 6 |
| Synthesis of results | 14 | Heterogeneity on mortality rate was assessed by using I^2^ statistics. For a more conservative approach, results of random effect models were presented. | 6 |

Page 1 of 2

| **Section/topic** | **#** | **Checklist item** | **Reported on page #** |
| --- | --- | --- | --- |
| Risk of bias across studies | 15 | The Begg-adjusted rank correlation test was used to test the presence of possible bias across studies. | 6 |
| Additional analyses | 16 | A sensitivity analysis was performed considering those studies reporting data on mortality rate after serum [Na+] improvement at 12 and 36 months of follow-up or after serum [Na+] ≥130 mmol/L at follow-up. A meta-regression analysis was performed to test the effect of age and serum [Na+] cut-off enrolment on overall mortality rate. In addition, a linear regression analysis model, weighting each study for the number of subjects enrolled, was performed to verify the independent effect of hyponatremia improvement on mortality after the adjustment for age and sex and serum [Na+] cut-off. | 6 |
| **RESULTS** | | |  |
| Study selection | 17 | Out of 335 retrieved articles, 320 articles were excluded for different reasons. The flow of the meta-analysis is summarized in Figure 1, and the characteristics of the trials included in the meta-analysis are summarized in Table 1. Among the 15 selected studies, 7 studies evaluated the effect of hyponatremia improvement on overall mortality rate in subjects with heart failure, 3 in hospitalized series of subjects, one each including patients subjected to orthopic liver transplantation, admitted to an emergency room or affected by lung cancer, myocardial infarction or pulmonary acute embolism (Table 1). | 7-8 |
| Study characteristics | 18 | The characteristics of the retrieved trials and the number of events recorded are reported in Tables 1. Retrieved trials included 12,797 hyponatremic subjects with a mean age of 66.1 years and a mean follow-up of 33.6 months. In particular, 14 studies enrolled only hyponatremic subjects, whereas in one case [25] a mixed cohort of hyponatremic/eunatremic patients was considered. | 7-8 |
| Risk of bias within studies | 19 | For a more conservative approach, results of random effect models were presented. | 7-8 |
| Results of individual studies | 20 | We included 1 forest plot of all studies (Figure 4) and 1 plot considering only those studies (n=8) reporting a threshold for serum [Na+] improvement >130 mmol/L (Figure 4). | 7-8 |
| Synthesis of results | 21 | Present results of each meta-analysis done, including confidence intervals and measures of consistency. | 7-8 |
| Risk of bias across studies | 22 | Funnel plot and Begg adjusted rank correlation test (Kendall’s τ: 0.00; p=1.00) suggested no major publication bias. Similar results were obtained by applying Egger’s regression (Intercept -1.24±0.79; p=0.14). | 7-8 |
| Additional analysis | 23 | Meta-regression analysis showed that the reduced mortality rate in patients in whom hyponatremia improved was more evident in older subjects (S=-0.07[-0.011;-0.03];p=0.002;I=4.11[1.42;7.41];p=0.004) and in those with lower serum [Na+] at enrollment (S=0.07[0.03;0.011];p=0.001; I=-9.05[-14.33;-3.78];p=0.001). The association between reduced mortality rate after serum [Na+] improvement and [Na+] cut-off at enrollment was confirmed in a multiple regression model even after adjustment for age, gender and follow-up (adj.r =0.640; p<0.0001). | 7-8 |
| **DISCUSSION** | | |  |
| Summary of evidence | 24 | This meta-analysis documents for the first time that improvement in serum [Na+] in hyponatremic patients is associated with a reduction in the increased mortality associated with this common electrolyte disorder. | 9-10-11 |
| Limitations | 25 | Several limitations should be recognized. First of all, the present data cannot clarify whether hyponatremia and/or its lack of correction contribute directly to poor outcomes or are simply markers for severity of underlying co-morbidities, and whether improvements of the underlying co-morbidities influenced both the serum [Na+] and the mortality of the hyponatremic patients. Accordingly, it should recognized that the data were adjusted only for age and gender, whereas the prevalence of associate morbidities was not considered as possible confounders, because they were not reported adequately in a sufficient number of studies. Hence, potential unmeasured confounders may have caused residual confounding effects, but the measured factors that are correlated with such confounders should have mitigated this bias. However, meta-analysis is particularly useful when there is a variety of reports with low statistical power; in this situation, pooling of data can improve power and provide a more convincing result. It should be also recognized that the duration of the available trials is relatively short. A further limitation is represented by incomplete reporting of the data on mortality rate in trials only marginally designed for the assessment of mortality endpoints after [Na+] improvement. In particular, all the data that were analyzed were from observational studies and none of them was originally designed to address clinical outcomes after serum [Na+] improvement. Accordingly, no placebo-controlled studies were available for inclusion in the present meta-analysis. Finally, the statistical analysis showed the presence of heterogeneity that was particularly evident in two studies (ref. 13 and 28). Finally, statistical analyses did not suggest any relevant publication bias, the possibility of selective reporting cannot be excluded. These considerations do not negate the value of the novel findings reported in our study, but rather highlight the need for additional, well-designed studies of clinical outcomes with effective therapies in patients with hyponatremia. | 10-11 |
| Conclusions | 26 | This meta-analysis documents for the first time that improvement in serum [Na+] in hyponatremic patients is associated with a reduction in the increased mortality associated with this common electrolyte disorder. | 11 |
| **FUNDING** | | |  |
| Funding | 27 | No separate funding was necessary for the undertaking of this systematic review and meta-analysis. |  |
