# Supplementary material for: Hyponatremia Improvement Is Associated with a Reduced Risk of Mortality: Evidence from a Meta-Analysis
Source: PLoS One. 2015 Apr 23;10(4):e0124105. doi: 10.1371/journal.pone.0124105 (PMC4408113; doi:10.1371/journal.pone.0124105)
Supplement: S2 File — (DOC) [file pone.0124105.s002.doc]

**Full-text articles that were excluded:**

1. **No mortality data (n=12):**

Prakoso E, Jones C, Koorey DJ, Strasser SI, Bowen D, McCaughan GW, et al. Terlipressin therapy for moderate-to-severe hyponatraemia in patients with liver failure. Intern Med J. 2013; 43:240-246.

Abraham WT, Decaux G, Josiassen RC, Yagil Y, Kopyt N, Thacker HP,et al. HARMONY Study Group. Oral lixivaptan effectively increases serum sodium concentrations in outpatients with euvolemic hyponatremia. Kidney Int. 2012; 82:1215-1222.

Sahin OZ, Asci G, Kircelli F, Yilmaz M, Duman S, Ozkahya M, et al. The impact of low serum sodium level on mortality depends on glycemic control. Eur J Clin Invest. 2012; 42:534-540.

Ginès P, Wong F, Watson H, Milutinovic S, del Arbol LR, Olteanu D; HypoCAT Study Investigators. Effects of satavaptan, a selective vasopressin V(2) receptor antagonist, on ascites and serum sodium in cirrhosis with hyponatremia: a randomized trial. Hepatology. 2008; 48:204-213.

Ayus JC, Arieff AI. Chronic hyponatremic encephalopathy in postmenopausal women: association of therapies with morbidity and mortality. JAMA. 1999; 281:2299-2304.

Erasmus RT, Matsha TE. The frequency, aetiology and outcome of severe hyponatraemia in adult hospitalised patients. Cent Afr J Med. 1998; 44:154-158.

Madiba TE, Haffejee AA, Mokoena TR. Hyponatraemia--a prospective analysis of surgical patients. Afr J Surg. 1998; 36:78-81.

Sowunmi A. Hyponatraemia in severe falciparum malaria: a clinical study of nineteen comatose African children. Afr J Med Med Sci. 1996; 25:47-52.

Ellis SJ. Severe hyponatraemia: complications and treatment. QJM. 1995; 88:905-909.

Arieff AI, Ayus JC, Fraser CL. Hyponatraemia and death or permanent brain damage in healthy children. BMJ. 1992; 304:1218-1222.

Wattad A, Chiang ML, Hill LL. Hyponatremia in hospitalized children. Clin Pediatr (Phila). 1992; 31:153-157.

Sterns RH. Severe symptomatic hyponatremia: treatment and outcome. A study of 64 cases. Ann Intern Med. 1987; 107:656-664.

1. **No serum sodium improvement data (n=37):**

Neeff HP, Streule GC, Drognitz O, Tittelbach-Helmrich D, Spangenberg HC, Hopt UT, et al. Early mortality and long-term survival after abdominal surgery in patients with liver cirrhosis. Surgery. 2014; 1554:623-632.

Mohan S, Gu S, Parikh A, Radhakrishnan J. Prevalence of hyponatremia and association with mortality: results from NHANES. Am J Med. 2013; 126 12:1127-1137.

Nigwekar SU, Wenger J, Thadhani R, Bhan I. Hyponatremia, mineral metabolism, and mortality in incident maintenance hemodialysis patients: a cohort study. Am J Kidney Dis. 2013; 62: 755-762.

Crestanello JA, Phillips G, Firstenberg MS, Sai-Sudhakar C, Sirak J, Higgins R, et al. Does preoperative hyponatremia potentiate the effects of left ventricular dysfunction on mortality after cardiac surgery? J Thorac Cardiovasc Surg. 2013; 145:1589-1594.

Shchekochikhin DY, Schrier RW, Lindenfeld J, Price LL, Jaber BL, Madias NE. Outcome differences in community- versus hospital-acquired hyponatremia in patients with a diagnosis of heart failure. Circ Heart Fail. 2013; 6:379-386.

Hagino T, Ochiai S, Watanabe Y, Senga S, Saito M, Takayama Y, et al. Hyponatremia at admission is associated with in-hospital death in patients with hip fracture. Arch Orthop Trauma Surg. 2013; 133:507-511

Gankam-Kengne F, Ayers C, Khera A, de Lemos J, Maalouf NM. Mild hyponatremia is associated with an increased risk of death in an ambulatory setting. Kidney Int. 2013; 83:700-706.

Sato N, Gheorghiade M, Kajimoto K, Munakata R, Minami Y, Mizuno M, et al. ATTEND Investigators. Hyponatremia and in-hospital mortality in patients admitted for heart failure (from the ATTEND registry). Am J Cardiol. 2013; 111:1019-1025.

Albert NM, Nutter B, Forney J, Slifcak E, Tang WH. A randomized controlled pilot study of outcomes of strict allowance of fluid therapy in hyponatremic heart failure (SALT-HF).J Card Fail. 2013; 19:1-9.

Huang WY, Weng WC, Peng TI, Chien YY, Wu CL, Lee M, et al. Association of hyponatremia in acute stroke stage with three-year mortality in patients with first-ever ischemic stroke. Cerebrovasc Dis. 2012; 34:55-62.

Konishi M, Haraguchi G, Ohigashi H, Sasaoka T, Yoshikawa S, Inagaki H,et al. Progression of hyponatremia is associated with increased cardiac mortality in patients hospitalized for acute decompensated heart failure. J Card Fail. 2012; 18:620-625.

Sersté T, Gustot T, Rautou PE, Francoz C, Njimi H, Durand F, et al. Severe hyponatremia is a better predictor of mortality than MELDNa in patients with cirrhosis and refractory ascites. J Hepatol. 2012; 57:274-280.

Kang SH, Kim HW, Lee SY, Sun IO, Hwang HS, Choi SR, et al. Is the sodium level per se related to mortality in hospitalized patients with severe hyponatremia? Clin Nephrol. 2012; 77:182-187.

Tolouian R, Alhamad T, Farazmand M, Mulla ZD. The correlation of hip fracture and hyponatremia in the elderly. J Nephrol. 2012; 25:789-793.

Wong F, Watson H, Gerbes A, Vilstrup H, Badalamenti S, Bernardi M, et al. Satavaptan Investigators Group. Satavaptan for the management of ascites in cirrhosis: efficacy and safety across the spectrum of ascites severity. Gut. 2012; 6:108-116.

Baldasseroni S, Urso R, Orso F, Bianchini BP, Carbonieri E, Cirò A, et al. Relation between serum sodium levels and prognosis in outpatients with chronic heart failure: neutral effect of treatment with beta-blockers and angiotensin-converting enzyme inhibitors: data from the Italian Network on Congestive Heart Failure (IN-CHF database). J Cardiovasc Med (Hagerstown). 2011; 12 :723-731

Balling L, Schou M, Videbæk L, Hildebrandt P, Wiggers H, Gustafsson F; Danish Heart Failure Clinics Network. Prevalence and prognostic significance of hyponatraemia in outpatients with chronic heart failure. Eur J Heart Fail. 2011; 13:968-973.

Schou M, Valeur N, Torp-Pedersen C, Gustafsson F, Køber L. Plasma sodium and mortality risk in patients with myocardial infarction and a low LVEF. Eur J Clin Invest 2011; 41:1237-1244.

Moini M, Hoseini-Asl MK, Taghavi SA, Sagheb MM, Nikeghbalian S, Salahi H, et al. Hyponatremia a valuable predictor of early mortality in patients with cirrhosis listed for liver transplantation. Clin Transplant. 2011; 25:638-645.

Shapiro DS, Sonnenblick M, Galperin I, Melkonyan L, Munter G. Severe hyponatraemia in elderly hospitalized patients: prevalence, aetiology and outcome. Intern Med J. 2010; 40:574-580.

Wald R, Jaber BL, Price LL, Upadhyay A, Madias NE. Impact of hospital-associated hyponatremia on selected outcomes. Arch Intern Med. 2010; 170:294-302.

Dimopoulos K, Diller GP, Petraco R, Koltsida E, Giannakoulas G, Tay EL, et al. Hyponatraemia: A strong predictor of mortality in adults with congenital heart disease. Eur Heart J. 2009; 31:595-601.

Hsu HH, Chen YC, Tian YC, Chan YL, Kuo MC, Tang CC, et al. Role of serum sodium in assessing hospital mortality in cancer patients with spontaneous tumour lysis syndrome inducing acute uric acid nephropathy. Int J Clin Pract. 2009 ; 63:751-756.

Carey RG, Bucuvalas JC, Balistreri WF, Nick TG, Ryckman FR, Yazigi N. Hyponatremia increases mortality in pediatric patients listed for liver transplantation. Pediatr Transplant. 2009; 14:115-120.

Whelan B, Bennett K, O'Riordan D, Silke B. Serum sodium as a risk factor for in-hospital mortality in acute unselected general medical patients. QJM. 2009; 102:175-82.

Kim WR, Biggins SW, Kremers WK, Wiesner RH, Kamath PS, Benson JT, et al. Hyponatremia and mortality among patients on the liver-transplant waiting list. N Engl J Med . 2008; 359:1018-1026.

Milo-Cotter O, Cotter G, Weatherley BD, Adams KF, Kaluski E, Uriel N, et al. Hyponatraemia in acute heart failure is a marker of increased mortality but not when associated with hyperglycaemia. Eur J Heart Fail. 2008; 10:196-200.

Konstam MA, Gheorghiade M, Burnett JC Jr, Grinfeld L, Maggioni AP, Swedberg K, et al.; Efficacy of Vasopressin Antagonism in Heart Failure Outcome Study With Tolvaptan (EVEREST) Investigators. Effects of oral tolvaptan in patients hospitalized for worsening heart failure: the EVEREST Outcome Trial. JAMA. 2007; 297:1319-1331..

Gheorghiade M, Abraham WT, Albert NM, Gattis Stough W, Greenberg BH, O'Connor CM, et al. OPTIMIZE-HF Investigators and Coordinators. Relationship between admission serum sodium concentration and clinical outcomes in patients hospitalized for heart failure: an analysis from the OPTIMIZE-HF registry. Eur Heart J. 2007; 28:980-988.

Gill G, Huda B, Boyd A, Skagen K, Wile D, Watson I, et alCharacteristics and mortality of severe hyponatraemia--a hospital-based study. Clin Endocrinol (Oxf). 2006; 65:246-249.

Goldberg A, Hammerman H, Petcherski S, Nassar M, Zdorovyak A, Yalonetsky S, et al. Hyponatremia and long-term mortality in survivors of acute ST-elevation myocardial infarction. Arch Intern Med. 2006; 16:781-786.

Ruf AE, Kremers WK, Chavez LL, Descalzi VI, Podesta LG, Villamil FG. Addition of serum sodium into the MELD score predicts waiting list mortality better than MELD alone. Liver Transpl. 2005; 11:336-343.

Goldberg A, Hammerman H, Petcherski S, Zdorovyak A, Yalonetsky S, Kapeliovich M, et al. Prognostic importance of hyponatremia in acute ST-elevation myocardial infarction. Am J Med. 2004; 117:242-248.

Borroni G, Maggi A, Sangiovanni A, Cazzaniga M, Salerno F. Clinical relevance of hyponatraemia for the hospital outcome of cirrhotic patients. Dig Liver Dis. 2000; 32:605-610.

Miller M, Morley JE, Rubenstein LZ. Hyponatremia in a nursing home population..J Am Geriatr Soc. 1995; 43:1410-1413.

Singhi S, Prasad SV, Chugh KS. Hyponatremia in sick children: a marker of serious illness.

Indian Pediatr. 1994; 31:19-25.

Tang WW, Kaptein EM, Feinstein EI, Massry SG. Hyponatremia in hospitalized patients with the acquired immunodeficiency syndrome (AIDS) and the AIDS-related complex. Am J Med. 1993; 94:169-174.
